# Just another “Clever Hans”? Neural networks and FDG PET-CT to predict the outcome of patients with breast cancer

**DOI:** 10.1007/s00259-021-05270-x

**Published:** 2021-03-05

**Authors:** Manuel Weber, David Kersting, Lale Umutlu, Michael Schäfers, Christoph Rischpler, Wolfgang P. Fendler, Irène Buvat, Ken Herrmann, Robert Seifert

**Affiliations:** 1 Department of Nuclear Medicine, University of Duisburg-Essen and German Cancer Consortium (DKTK)-University Hospital, Hufelandstraße 55, 45147 Essen, Germany; 2 West German Cancer Center, Essen-Münster, Germany; 3 Department of Diagnostic and Interventional Radiology and Neuroradiology, University Hospital Essen, University Duisburg-Essen, Essen, Germany; 4 Laboratoire d’Imagerie Translationnelle en Oncologie, Inserm, Institut Curie, Université Paris Saclay, Orsay, France; 5 Department of Nuclear Medicine, University Hospital Münster, University Münster, Münster, Germany

**Keywords:** Neural network, Metabolic tumor volume, ^18^F-FDG PET, Breast cancer

## Abstract

**Background:**

Manual quantification of the metabolic tumor volume (MTV) from whole-body ^18^F-FDG PET/CT is time consuming and therefore usually not applied in clinical routine. It has been shown that neural networks might assist nuclear medicine physicians in such quantification tasks. However, little is known if such neural networks have to be designed for a specific type of cancer or whether they can be applied to various cancers. Therefore, the aim of this study was to evaluate the accuracy of a neural network in a cancer that was not used for its training.

**Methods:**

Fifty consecutive breast cancer patients that underwent ^18^F-FDG PET/CT were included in this retrospective analysis. The PET-Assisted Reporting System (PARS) prototype that uses a neural network trained on lymphoma and lung cancer ^18^F-FDG PET/CT data had to detect pathological foci and determine their anatomical location. Consensus reads of two nuclear medicine physicians together with follow-up data served as diagnostic reference standard; 1072 ^18^F-FDG avid foci were manually segmented. The accuracy of the neural network was evaluated with regard to lesion detection, anatomical position determination, and total tumor volume quantification.

**Results:**

If PERCIST measurable foci were regarded, the neural network displayed high per patient sensitivity and specificity in detecting suspicious ^18^F-FDG foci (92%; CI = 79–97% and 98%; CI = 94–99%). If all FDG-avid foci were regarded, the sensitivity degraded (39%; CI = 30–50%). The localization accuracy was high for body part (98%; CI = 95–99%), region (88%; CI = 84–90%), and subregion (79%; CI = 74–84%). There was a high correlation of AI derived and manually segmented MTV (*R*^2^ = 0.91; *p* < 0.001). AI-derived whole-body MTV (HR = 1.275; CI = 1.208–1.713; *p* < 0.001) was a significant prognosticator for overall survival. AI-derived lymph node MTV (HR = 1.190; CI = 1.022–1.384; *p* = 0.025) and liver MTV (HR = 1.149; CI = 1.001–1.318; *p* = 0.048) were predictive for overall survival in a multivariate analysis.

**Conclusion:**

Although trained on lymphoma and lung cancer, PARS showed good accuracy in the detection of PERCIST measurable lesions. Therefore, the neural network seems not prone to the clever Hans effect. However, the network has poor accuracy if all manually segmented lesions were used as reference standard. Both the whole body and organ-wise MTV were significant prognosticators of overall survival in advanced breast cancer.

**Supplementary Information:**

The online version contains supplementary material available at 10.1007/s00259-021-05270-x.

## Introduction

The staging of patients with various malignancies is routinely carried out by ^18^F-FDG PET/CT based on qualitative visual analyses by trained medical professionals [1–4]. The tumor burden can also be assessed quantitatively, by measuring the whole-body metabolic tumor volume (MTV) from ^18^F-FDG PET/CT. Yet, this requires laborious tumor delineation and is thus generally omitted in the clinical routine. This causes a clinical issue, as MTV is of increasing importance for outcome prognostication and measurement of treatment response [5–7], in particular in breast cancer [7]. In contrast, rudimentary simplified staging systems like the Deauville score or PERCIST are used as surrogates [8, 9].

Neural networks have shown human-like performance on well-defined tasks including medical imaging analysis and might thereby provide assistance to medical professionals [10–14]. A recently published study demonstrated high accuracy of a neural network for the identification of suspicious (i.e., malignant) ^18^F-FDG PET/CT foci in lymphoma and lung cancer patients [15].

The vision of automated whole-body MTV quantification might become reality thanks to the PARS (PET-Assisted Reporting System) investigational software prototype, which among others employs neural networks to identify lesions with suspicious FDG. However, as PARS was trained and validated on lymphoma and lung cancer patients only, the accuracy of the PARS in reading ^18^F-FDG PET/CTs of other tumor entities is uncertain. The neural networks of PARS could have learned strategies that are specifically useful for the tumor detection in lymphoma and lung cancer, but these strategies might not be appropriate for other entities like breast cancer. This could be explained by the “Clever Hans” effect: a neural network that is capable of accomplishing a posed task may cheat by using unallowed information (e.g., detection of an ileus by nasogastric tube instead of dilated intestine, or in the present case, regarding only uptake in the lung and lymph node stations and rate is per se as suspicious) [16, 17]. The effect is named after the horse Hans that was believed to perform math calculations but was actually well trained at reading the emotions of the auditor without any skills in math [17].

The aim of the present study was therefore to evaluate the accuracy of the PARS prototype when analyzing ^18^F-FDG PET/CT acquisitions of patients with breast cancer, a cancer entity with a different metastatic spread pattern that was not used for the training of the neural network embedded in PARS. To this end, per-patient and per-lesion detection rate and MTV quantification were recorded. Consensus reads by two nuclear medicine experts were used as reference standard. Additionally, the prognostic value of manually and neural network-derived MTV with regard to overall survival was evaluated. Thus, the applicability of the PARS prototype was explored, and insights on the design of future neural networks for the reading of PET images were obtained.

## Methods

### Patients

We retrospectively screened our institutional FDG-PET/CT database for 50 consecutive patients with breast cancer and follow-up for ≥5 years or until death. Patients referred to ^18^F-FDG PET/CT by the West German Cancer Center were consecutively included in this analysis. Thereby, detailed patient characteristics as well as follow-up data are present (see Table 1).

**Table 1:** Patient characteristics

Category		
Sex	Female, *n* (%)	50 (100)
	Male, *n* (%)	0 (0)
Age	Mean (years)	56.7
Histopathology	IDC, *n* (%)	25 (50.0)
	ILC, *n* (%)	3 (6.0)
	IMC, *n* (%)	1 (2.0)
	PBC, *n* (%)	1 (2.0)
	N/A, *n* (%)	20 (40.0)
	Estrogen-pos, *n* (%)	27 (54.0)
	Estrogen-neg, *n* (%)	10 (20.0)
	Estrogen-N/A, *n* (%)	13 (26.0)
	Progesterone-pos, *n* (%)	19 (38.0)
	Progesterone-neg, *n* (%)	18 (36.0)
	Progesterone-N/A, *n* (%)	13 (26.0)
	HER2/neu-pos, *n* (%)	8 (16.0)
	HER2/neu-neg, *n* (%)	29 (58.0)
	HER2/neu-N/A, *n* (%)	13 (26.0)
UICC Stage	I (%)	A, 3 (6%); B, 1 (2%)
	II (%)	A, 4 (8%)
	III (%)	C, 2 (4%)
	IV (%)	40 (80%)
Imaging characteristics	Activity, mean ± SD	253.9 ± 52.0
	Uptake time, mean ± SD	61.1 ± 11.6
	CE CT, *n* (%)	48 (96.0)
Follow-up	Deceased, *n* (%)	33 (66.0)
	Mean OS (months)	43.9

*UICC* Union for International Cancer Control, *n* number, *IDC* invasive-ductal carcinoma, *ILC* invasive lobular carcinoma, *IMC* invasive mucinous carcinoma, *PBC* papillary breast cancer, *N/A* not available, *pos* positive, *neg* negative, *SD* standard deviation, *CE* contrast-enhanced, *CT* computed tomography, *OS* overall survival

### PET imaging

A Siemens Biograph mCT system was used for image acquisitions (Siemens Healthineers, Knoxville, TN, USA). ^18^F-FDG was injected, if blood glucose levels were < 200 mg/dl following EANM procedure guidelines for FDG-PET/CT in tumor imaging [18]. Image acquisition was initiated mean (±SD) 61.1 (±11.6) min after the administration of a mean (±SD) activity of 253.9 (±52.0) MBq ^18^F-FDG. The field of view comprised vertex to proximal femur. Contrast-enhanced CT was available in 48/50 (96%) cases. PET image reconstruction was done using the ordered subset expectation maximization algorithm (voxel size 3.18 × 3.18 × 5.0 mm, 3 iterations, 24 subsets).

### Manual reading as reference standard for the neural network

A semi-automated thresholding approach was used to assist the segmentation of pathological FDG-avid foci. Small foci (MTV < 0.5 ml) were neglected. A mediastinal blood pool-specific (therefore patient specific) threshold (2 x mean (SUV) + 2 x std. (SUV)) was used in analogy to PERCIST criteria to preselect FDG-avid foci [9]. FDG-avid foci unrecognized by this procedure were then added manually. Thereafter, the anatomical location (see Table 2) and classification (suspicious, i.e., pathological or unsuspicious, i.e., physiological) were manually determined for each of the 1072 segmented foci by M.W. and D.K. in consensus reading. Body part (e.g., thorax, abdomen) and region (usually an organ, e.g., spleen, bones) were specified for all lesions, but subregion (e.g., spine, lymph node level) was not. For example, a focus in the spleen does only have a body part (i.e., abdomen) and region (i.e., spleen) but not a subregion label. To compare the accuracy of the anatomical labels, the most detailed anatomical classification refers to the finest manually provided anatomical classification of a focus, regardless if this was on the region or subregion level.

**Table 2:** Anatomical locations that were employed by the expert readers and neural network

	Anatomical label
Body part	Cranium, neck, abdomen, upper limb, lower limb.
Region	E.g., esophagus, lung, pleura, heart, thymus, mediastinum, bones, skin, muscles, breast, liver
Subregion	E.g., scapula, sternum, lymph node IASLC station 1, mesenterial lymph nodes

This consensus read served as reference standard to assess the accuracy of the neural network in detecting suspicious FDG-avid foci. Suspicious FDG-avid foci were classified as measurable according to PERCIST criteria (SUVpeak >2 × mean (SUV liver) + 2 × std. (SUVliver)) [9]. The metabolic tumor volume was quantified by relative thresholding (50% of local SUV_max_).

### Neural network for automated identification of suspicious ^18^F-FDG foci

**Fig. 1:**
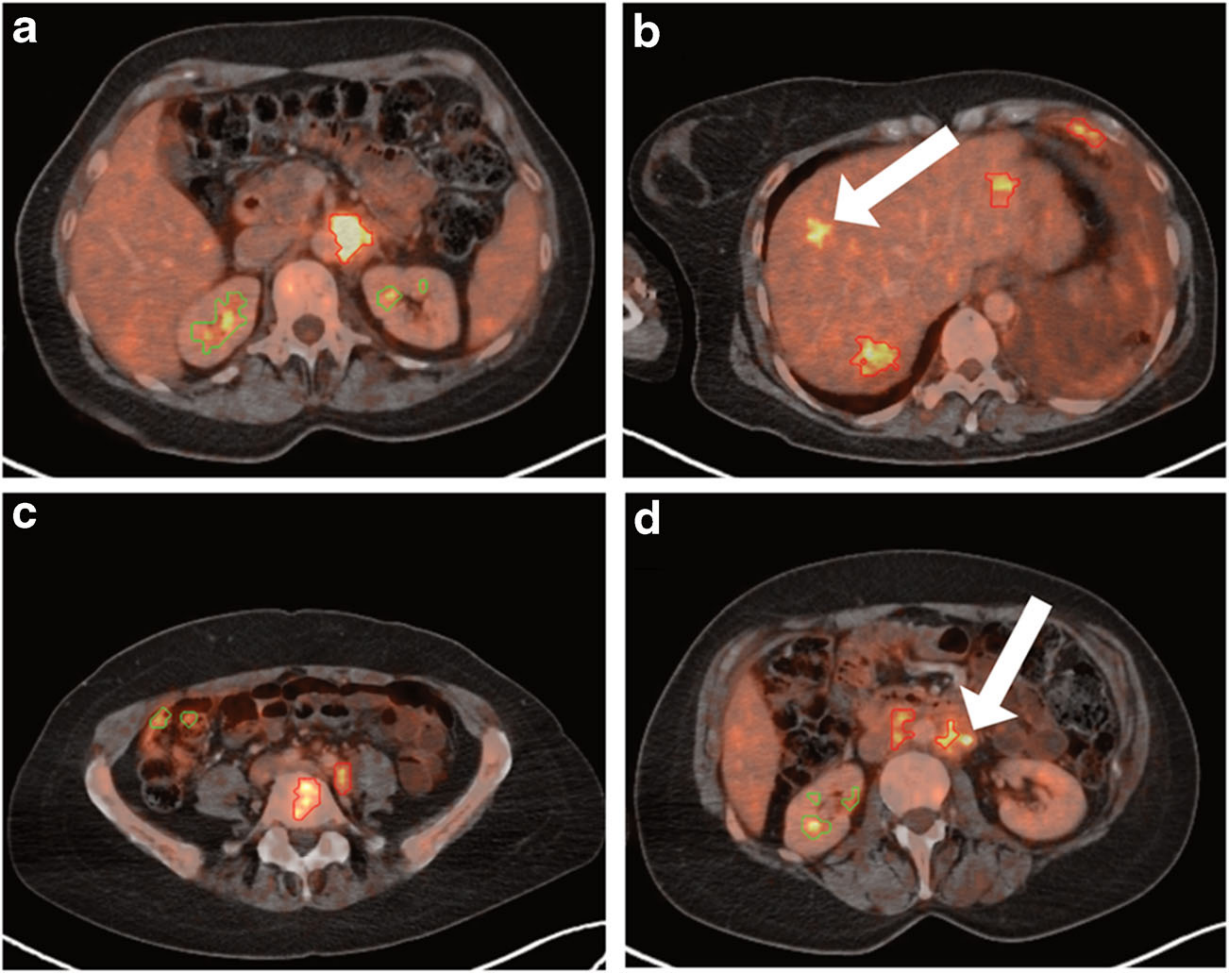
Exemplary automated classification by neural network. Axial FDG PET/CT images are shown of an exemplary patient. Segmentation and lesion classification were done by the neural network. Physiological uptake is marked in green, pathological uptake in red. The white arrows mark foci that were missed by the neural network

A research prototype implementing a neural network (PET-Assisted Reporting System, PARS v3.0) was used under a research license agreement for the fully automated identification of suspicious ^18^F-FDG-avid foci (Siemens Medical Solutions USA, Inc., Knoxville, TN, USA) [15]. PARS used a two-step procedure: First, ^18^F-FDG-avid foci were determined by automated thresholding of the PET. For thresholding, the same settings as for the expert raters were chosen, which found both pathologically and physiologically caused ^18^F-FDG foci (see above). Second, PARS classified each focus as unsuspicious or suspicious. Additionally, the anatomical position of each focus was determined. An exemplary finding of PARS is given in Fig. 1. Lesions identified by PARS were compared to manually identified lesions. For each manually identified lesion, it was checked if the lesion was identified by PARS as well. Additionally, the anatomical label of each lesion was compared between PARS and manual reads.

### Statistical analysis

MATLAB 2019b (The MathWorks, MA, USA) and Excel (Microsoft, WA, USA) were used for data handling. SPSS v25 (IBM, NY, USA) was used for regressions; Wilcoxon signed-rank test and Mann–Whitney *U* test. Logarithmic transformation was done for Cox and Pearson regression to correct for skewness of data (base 2; 1 was added to all values prior to transformation) [19]. For stepwise Cox regression, the forward LR method of SPSS was used with standard settings. Gönen and Heller’s concordance index was employed to measure the accuracy of the Cox regression; a bootstrap Gauss test was employed to test for statistical differences [15, 20, 21]. R v3.5.2 (The R Foundation, r-project.org) was used for bootstrapping, descriptive accuracy metrics, and Kaplan Meier plotting. Ninety-five percent confidence intervals (95% CI) of accuracy metrics were determined by bootstrapping with 1000 replicates. For the patient and focus-wise analysis, resampling was done on the patient level, as in [15], as the FDG foci of a given patient were not statistically independent from each other. This minimizes confounding effects possibly introduced by patients with many lesions with high SUV values that are easier to interpret by PARS.

## Results

### Identification and classification of suspicious FDG foci

In total, the location and classification of 1072 ^18^F-FDG avid foci were examined; as determined by consensus reads, 499 foci were caused by physiological tracer uptake; 573 foci were caused by pathological uptake. The mean number of foci per patient was 21 (range 3–68). Of the 1072 foci, 322 were manually added. The sensitivity of the neural network in the identification and classification of suspicious ^18^F-FDG-foci was 47% (95% CI 38–56%) per focus and 39% (95% CI 30–50%) in mean per patient. There were 768 PERCIST measurable findings (477 benign and 291 suspicious). The mean number of PERCIST measurable findings per patient was 15 (range 2–55). If only PERCIST measurable ^18^F-FDG-foci were regarded, the sensitivity for suspicious foci was 92% (95% CI 89–95%) per focus and 92% (95% CI 79–97%) in mean per patient. The MTV of manually added lesions was significantly smaller compared to not missed lesions; the same was true for SUV_max_ (1.3 vs. 3.6 ml; *p* < 0.001|4.7 vs. 8.3 SUV; *p* < 0.001).

The identification and classification accuracy of PARS in rating ^18^F-FDG-avid foci as suspicious or unsuspicious was 70% (95% CI 64–76%) per focus and 72% (95% CI 65–77%) in mean per patient. If only PERCIST measurable foci were regarded, the detection and classification accuracy was 96% (95% CI 94–97%) per focus and 97% (95% CI 96–99%) in mean per patient. Table 3 additionally shows specificity, positive predictive value, and negative predictive value per focus and per patient of PERCIST measurable suspicious ^18^F-FDG foci. Supplementary Table 1 shows these metrics for all foci.

**Table 3:** Identification and classification of suspicious FDG-avid foci by fully automated neural network when compared with the consensus reader reference standard

	Per lesion	Per patient
Accuracy	95.6 (93.9–97.2) %	97.3 (95.7–98.5) %
Sensitivity	92.1 (89.3–94.8) %	92.3 (79.3–96.6) %
Specificity	97.7 (95.0–98.9) %	97.6 (94.2–99.1) %
Positive predictive value	96.1 (91.4–98.2) %	96.5 (92.1–98.6) %
Negative predictive value	95.3 (90.8–97.4) %	93.2 (86.2–97.1) %

Only PERCIST measurable lesions were regarded for this table

If only PERCIST measurable foci were regarded, classification accuracy in the region of the primary tumor (pectoral muscle or breast) was 67% (95% CI 50–100%) per focus lesion. If all lesions were regarded (50 foci), the detection and classification accuracy decreased to 38% (95% CI 25–100%).

### Accuracy of neural network-based anatomical label determination

The accuracy of anatomical label classification per ^18^F-FDG focus was 98% (95% CI 95–99%) per body part, 88% (95% CI 84–90%) per region, and 80% (95% CI 74–84%) per subregion. The per-patient accuracy of anatomical label classification of FDG foci was 98% (95% CI 97–99%) per body part, 88% (95% CI 84–90%) per region, and 79% (95% CI 72–84%) per subregion. The most detailed anatomical classification of a given focus was determined with an accuracy of 84% (95% CI 81–87%) on a focus level and 85% (95% CI 81–88%) in mean per patient.

### Metabolic tumor volume

**Fig. 2:**
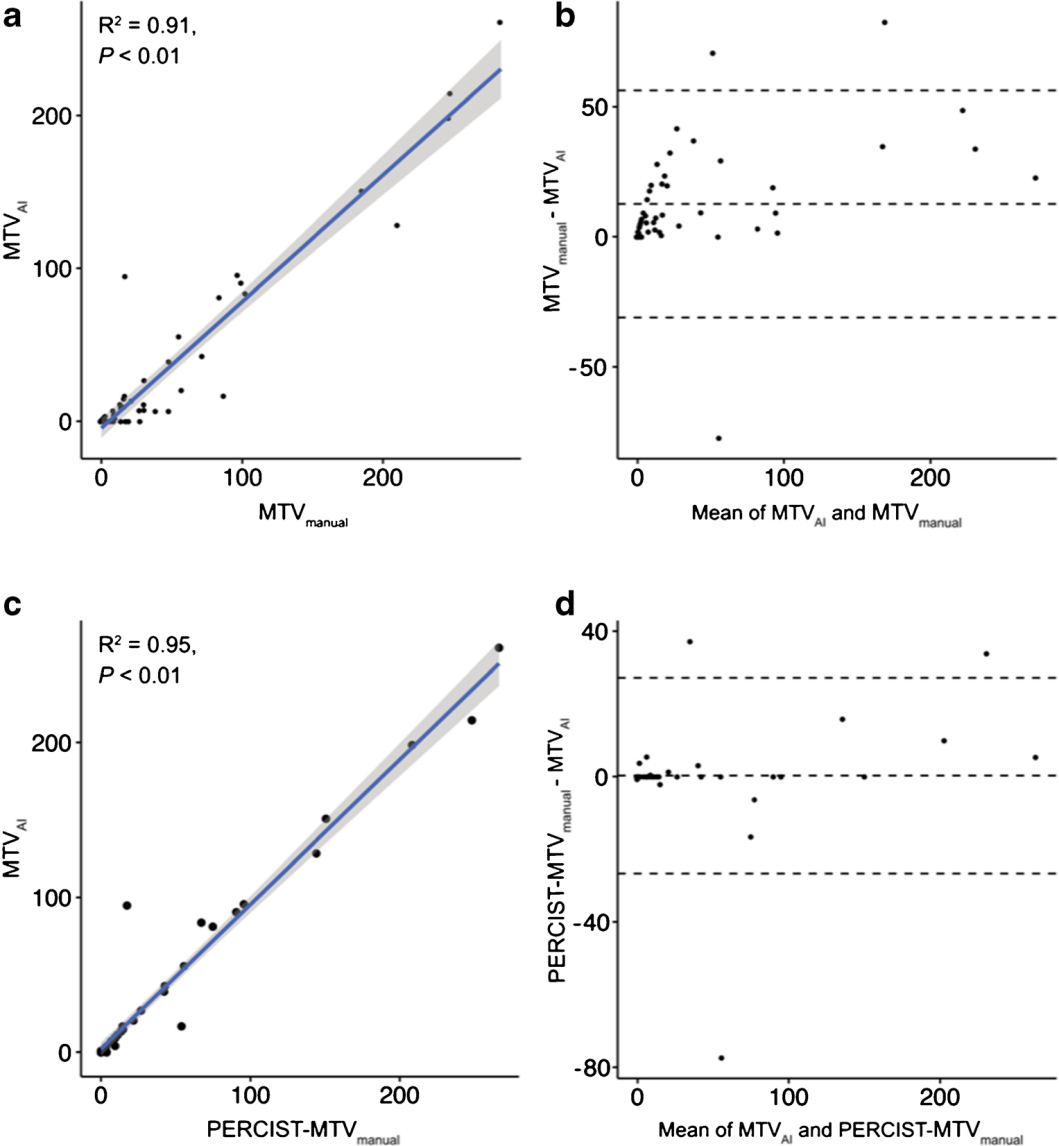
Correlation and Bland-Altman plots of metabolic tumor volume (in mL). Plots were shown for MTV_AI_ and MTV_manual_ (**a**, **b**) and for MTV_AI_ and PERCIST-MTV_manual_ (**c**, **d**). PERCIST-MTV_manual_ denotes the MTV of lesions that were measurable according to PERCIST

The automatically derived metabolic tumor volume (MTV_AI_) was statistically significantly smaller when compared to the manually determined metabolic volume (MTV_manual_) (median 7.0 vs. 17.3 ml, *p* < 0.001). There was a statistically significant correlation between MTV_AI_ and MTV_manual_ (*R*^2^ = 0.91; *p* < 0.001; Fig. 2). If only PERCIST measurable suspicious foci were regarded, there was no statistically significant difference between MTV_AI_ and MTV_manual_ (median 7.0 vs. 7.3 ml, *p* = 0.330).

**Fig. 3:**
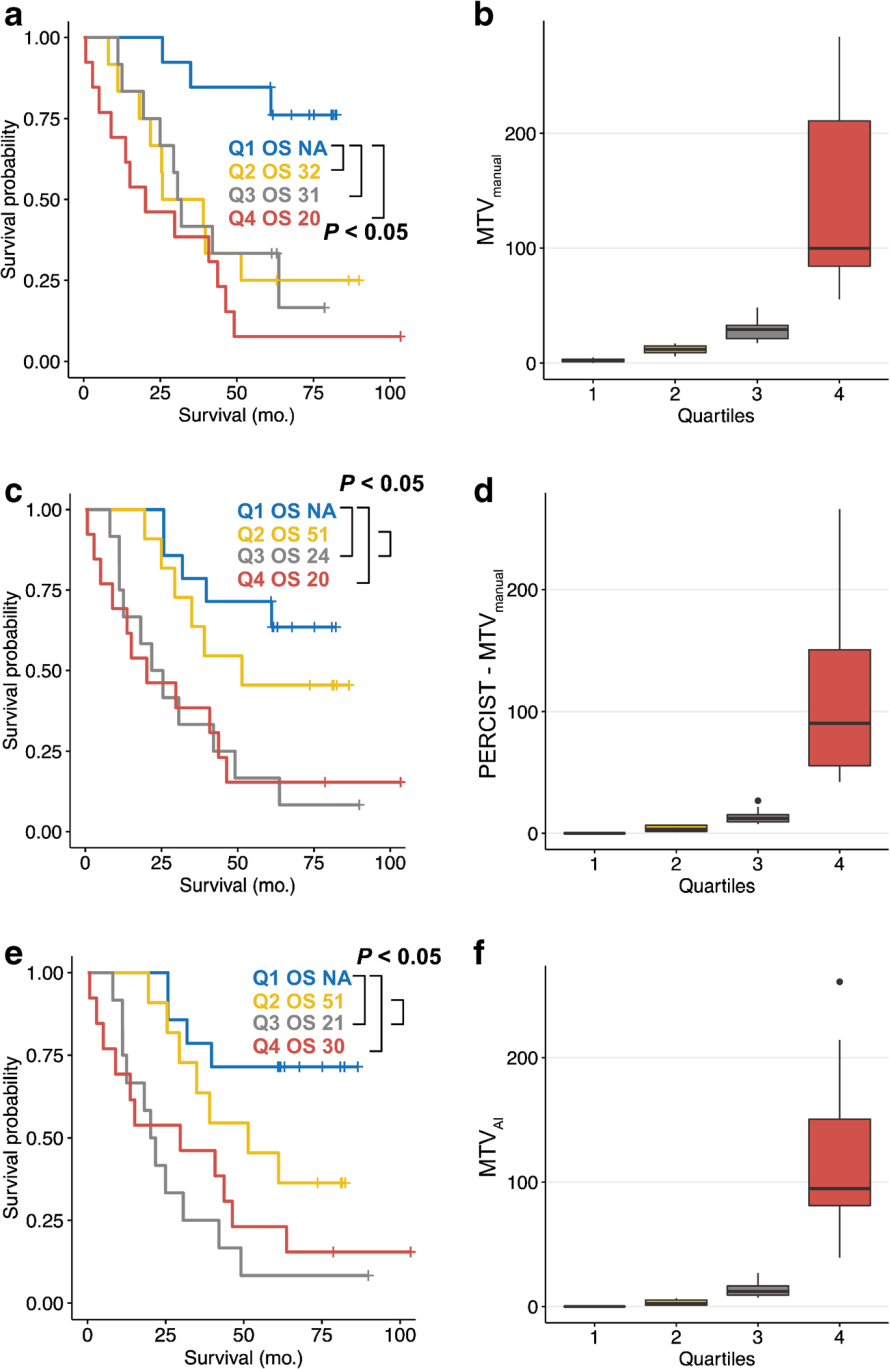
Overall survival and whole-body tumor volume. Kaplan Meier plots and boxplots are shown for the quartiles of MTV_manual_ (**a**, **b**), PERCIST-MTV_manual_ (**c**, **d**), and MTV_AI_ (**e**, **f**). For each quartile, median overall survival time (OS) is given in months

Log-transformed MTV_AI_ was a statistically significant prognosticator of overall survival time (HR = 1.275; 95% CI = 1.122–1.448; *p* < 0.001). The same was true for log-transformed MTV_manual_ (HR = 1.438; 95% CI = 1.208–1.713; *p* < 0.001). Kaplan Meier plots of MTV_manual_, PERCIST-MTV_manual_, and MTV_AI_ quartiles are shown by Fig. 3. Gönen and Heller’s concordance as marker for Cox model goodness of fit was not statistically significantly different between MTV_manual_ and PERCIST-MTV_manual_ (0.69 vs. 0.68; *p* = 0.30), MTV_AI_ and PERCIST-MTV_manual_ (0.65 vs. 0.68; *p* = 0.39), and MTV_AI_ and MTV_manual_ (0.65 vs. 0.69; *p* = 0.19).

### Organ-wise metabolic tumor volume

**Fig. 4:**
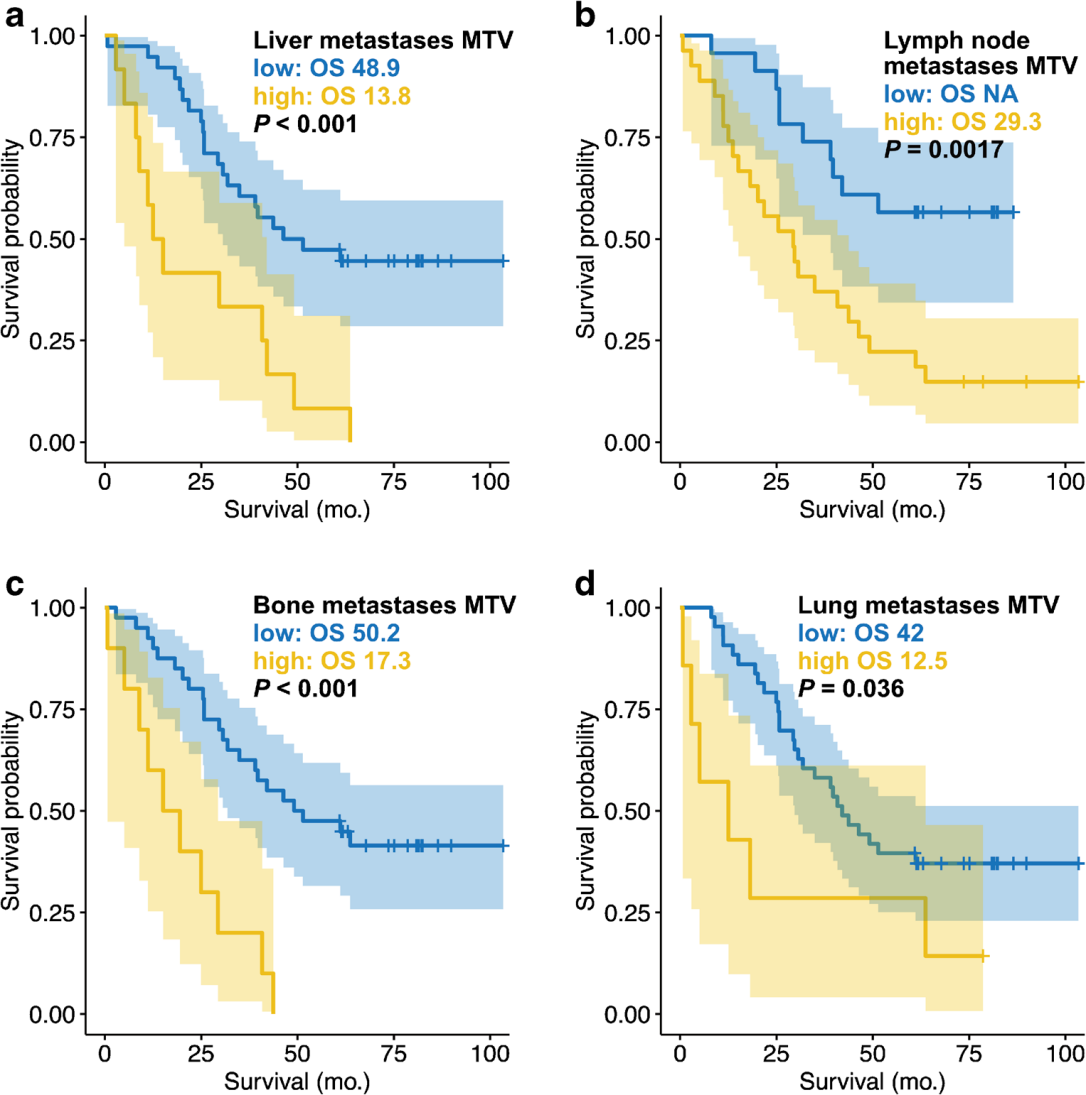
Overall survival and organ system wise tumor volume. The organ-wise MTV_AI_ is shown by Kaplan Meier plots (**a** liver metastases, **b** lymph node metastases, **c** bone metastases, and **d** lung metastases). Median overall survival time (OS) is given in months. The binarization in low and high was done by an optimized log rank cutoffs: liver-MTV_AI_ (0 ml), lymph node-MTV_AI_ (0 ml), bone-MTV_AI_ (2.1 ml), and lung-MTV_AI_ (0 ml)

Only liver-MTV_manual_ (HR = 1.178; 95% CI = 1.025–1.355; *p* = 0.021) and lymph node-MTV_manual_ (HR = 1.266; 95% CI = 1.081–1.482; *p* = 0.003) remained statistically significant prognosticators of overall survival in a multivariate stepwise Cox regression that additionally included bone-MTV_manual_, lung-MTV_manual_, and soft tissue-MTV_manual_ as covariates. Likewise, liver-MTV_AI_ (HR = 1.149; 95% CI = 1.001–1.318; *p* = 0.048) and lymph node-MTV_AI_ (HR = 1.190; 95% CI = 1.022–1.384; *p* = 0.025) were statistically significant prognosticators of overall survival in a multivariate regression. Table 4 presents the difference between MTV_AI_ and MTV_manual_ separately for bone, lymph node, liver, lung, and soft tissue foci that were rated as suspicious. Figure 4 displays idealized cutoff values for liver-MTV_AI_ (0 ml), lymph node-MTV_AI_ (0 ml), bone-MTV_AI_ (2.1 ml), and lung-MTV_AI_ (0 ml) by Kaplan Meier plots.

**Table 4:** Tumor volume per organ system

	MTV_AI_	MTV_manual_	*P* value
Bone	7.4 (0.0–236.7) ml	11.7 (0.0–256.1) ml	<0.001
Lymph node	12.6 (0–108.5) ml	14.0 (0–115.6) ml	0.03
Liver	9.2 (0.0–196.4) ml	9.3 (0.0–230.3) ml	0.557
Lung	1.9 (0.0–43.5) ml	5.9 (0.0–113.6) ml	0.015
Soft tissue	4.4 (0.0–105.2) ml	5.5 (0.0–85.2) ml	0.015

Values are presented as mean together with range

## Discussion

The present study evaluated the diagnostic accuracy of the PARS prototype to fully automatically determine the whole-body MTV of breast cancer patients. Despite the fact that the neural network involved in PARS was not trained on breast cancer ^18^F-FDG PET/CT data, both the identification of suspicious ^18^F-FDG foci and their anatomical location determination showed a high accuracy if limited to PERCIST measurable foci. Thereby, the whole-body and organ-wise MTV could be automatically determined with great accuracy. Whole-body and organ-wise MTV were significant prognosticators of overall survival time in advanced breast cancer patients.

Recently, the PARS prototype was proposed, which fully automatically segments foci with high uptake in ^18^F-FDG PET/CT and fully automatically determines which foci are suspicious [15]. PARS uses nine coronal reformatted 120 × 120-mm PET and CT slices of lesions with increased FDG uptake, a coronal maximum intensity projection reformatted PET data, and coordinates in an atlas space as input and classifies each foci as either benign or suspicious [15]. This neural network was designed and evaluated using PET acquisitions of lymphoma and lung cancer patients. It seems obvious that the network might be employed for ^18^F-FDG PET/CT tumor volume quantification of all cancer entities. Yet, neural networks and artificial intelligence are often referred to as black box, which is due to the fact that one cannot decipher the process of decision making [22]. Therefore, it is of great clinical importance to evaluate the performance of neural networks on tasks they were not explicitly trained for, but that are analogous to the original training task, to characterize their generalizability. In particular, it is essential to rule out the Clever Hans effect, which occurs when neural networks employ spurious correlation for their decision making [16]. For example, the neural network might have memorized the pattern of pathological FDG foci present in lung cancer and lymphoma due to their anatomical location. ^18^F-FDG PET/CT foci in the lung or in lymph node stations might have per se been rated as malign by PARS, which could suggest high segmentation accuracy. Thus, the performance of the PARS prototype was evaluated on ^18^F-FDG PET/CT of breast cancer patients in the present study.

To our knowledge for the first time, the performance of the PARS prototype was examined on ^18^F-FDG PET/CT scans of breast cancer patients in the present study. Disease patterns that are quasi-exclusive to breast cancer patients, such as the primary tumor lesion(s) or pectoral muscle infiltration, might have been erroneously missed by the PARS neural network. Therefore, the accuracy in this region was analyzed in a sub analysis. Indeed, we could show that the lesion detection accuracy in the pectoral region is lower compared to the overall accuracy. However, the overall detection accuracy of suspicious FDG foci was remarkably high in patients with advanced breast cancer. Yet, this is only true if FDG-avid foci were regarded that fulfill the PERCIST measurability requirement. Detection sensitivity and specificity of PERCIST measurable lesions were comparable to the lung cancer results of the initial PARS publication (sensitivity: lung cancer 87% vs. breast cancer 92%, specificity: lung cancer 99%, breast cancer 98%) [15]. The same was true for the location accuracy (body part: lung cancer 97% vs. breast cancer 98%; region: lung cancer 84% vs. breast cancer 88%) [15]. The higher accuracy in the breast cancer dataset might partially be explained by the fact that only a Biograph mCT was used for acquisition instead of a combination of the Biograph mCT and the older Biograph 16 [15]. As the metastatic patterns of lymphoma and lung cancer on the one hand and breast cancer on the other are different from each other, the high accuracy in the detection of suspicious FDG foci in breast cancer patients was not obvious. This finding indicates that neural networks might be suitable for the reading of ^18^F-FDG PET/CTs of patients with malignancies other than the ones they were originally trained for. Yet, a neural network aiming to automatically read ^18^F-FDG PET/CT data might profit from including primary staging examinations of various cancer entities in its training to ensure that the network is accurate in more anatomical locations.

**Fig. 5:**
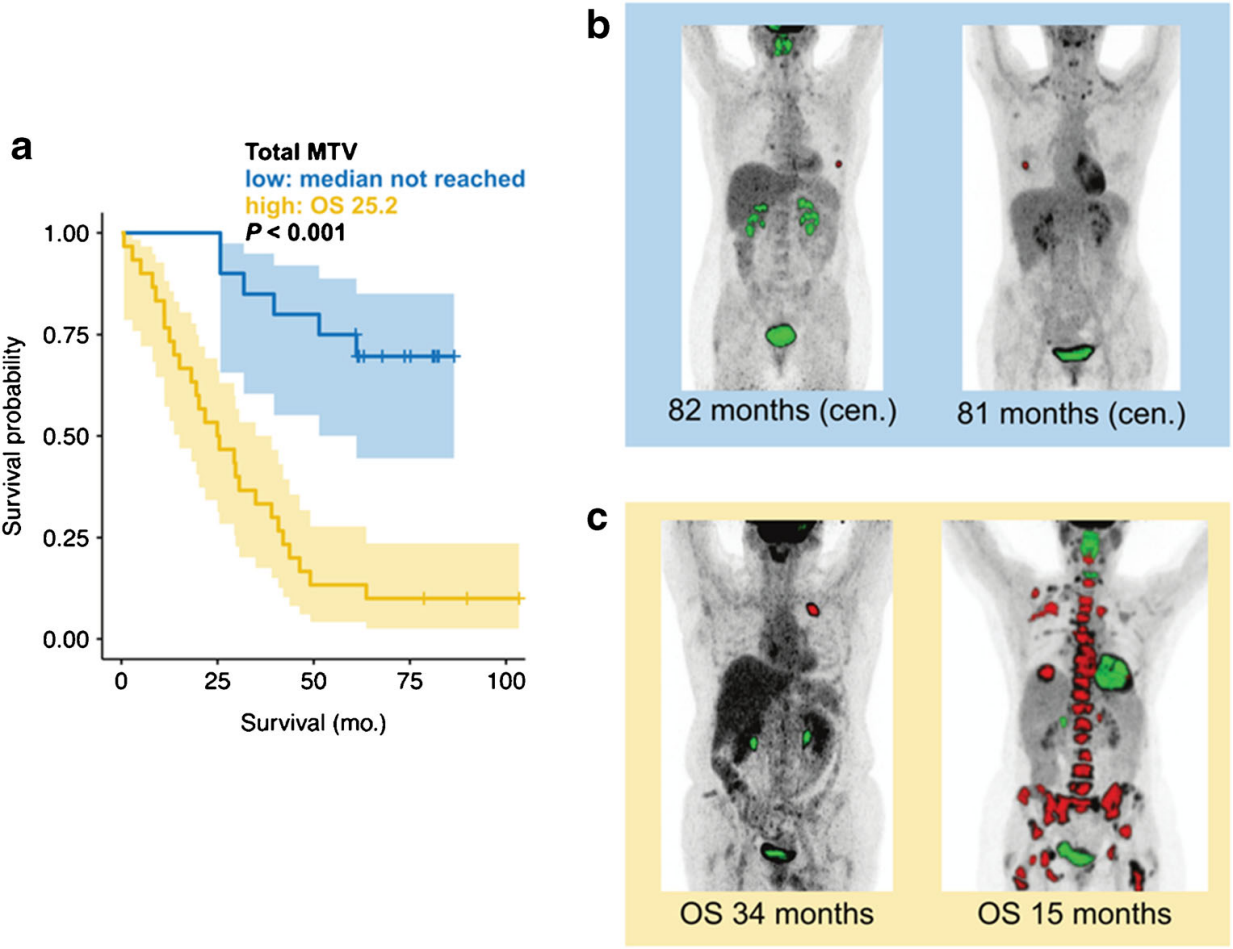
Overall survival and total MTV_AI_. Patients with a total MTV_AI_ smaller than 2.3 ml have significantly longer survival compared to those with greater MTV_AI_ (**a**). Overall survival (OS) is shown as median survival time in months (**a**) or in actual survival time from time of PET till death (**c**). Two exemplary cases of patients with low (**b**) and high MTV_AI_ (**c**) were shown; additionally, physiological FDG uptake is marked in green, pathological in red. Patients shown in panel **b** have not deceased (cen., censored). Note that patients with visually similar MTV_AI_ (**b** right image and **c** left image) show different outcomes and were grouped in the low and high MTV_AI_ groups respectively

Over the last years, a growing body of evidence has shown the predictive potential of MTV derived from ^18^F-FDG PET/CT in a variety of tumor types [5–7, 23, 24]: MTV has emerged as a biomarker in metastatic breast cancer after neoadjuvant chemotherapy independent from histopathologic subtype or tumor stage and allows for better risk stratification than conventional standardized uptake value (SUV) measurements [7]. Even small changes in MTV might have a considerable impact on the risk of poor outcome (Fig. 5). However, the segmentation of a whole-body MTV is generally not performed in the clinical routine. This is due to the fact the delineation of all tumor foci is too time consuming and insufficiently standardized. Rather simplified systems like Deauville or PERCIST are used to monitor the treatment response and to profile the risk of patients [8, 9]. Thereby, only few tumor lesions are regarded, and the majority of tumor lesions is discarded. However, due to metastatic heterogeneity, the response of tumor lesions might be discordant, which is not adequately covered by the quantification of a few target lesions [25, 26]. This clinical demand might be addressed by neural networks like the one in PARS to automatically segment the whole-body MTV. To date, neural networks for the fully automated quantification of the ^18^F-FDG PET/CT whole-body tumor volume have not been evaluated for malignancies aside from lymphoma [27], especially not for breast cancer. It was first shown by the present study that fully automatically derived whole-body MTV is a significant prognosticator of overall survival in breast cancer patients.

In the present study, manually and automatically derived whole-body MTV were correlated to a high degree. Moreover, the organ-wise MTV was well correlated between automated and manual delineation. Neural network derived organ wise MTV has not been studied before. The organ-wise MTV is clinically demanded, as it is known that the metastasis location has a profound impact on the outcome of the patients. This is partly explained by the fact that genomic alterations are present between the primary tumor and each metastatic side [25, 26]. In breast cancer, liver metastases are associated with worse outcome, compared to other metastatic sites like the skeleton [28]. This is corroborated by the present study, as the automatically determined MTV of lymph node and liver were statistically significant prognosticators of overall survival. The manual segmentation of organ system-wise MTV is even more time consuming compared to the whole-body MTV, as the reader has to annotate the location of every segmented metastasis. Therefore, neural networks like the one in PARS are needed to assist the nuclear medicine expert in the clinical routine.

The PARS prototype uses conventional thresholding and subsequently classifies found ^18^F-FDG foci as malign or suspicious. This network design achieved high accuracy in lymphoma and lung cancer patients. In this study, we could show that the accuracy is likewise high in breast cancer, if only PERCIST measurable foci were regarded [9]. However, the accuracy heavily decreased if smaller FDG-avid foci were regarded as well. Therefore, future neural networks should incorporate FDG-avid foci segmentation and not rely on conventional thresholding, which per se neglects foci of a given activity. Especially for the tumor volume quantification of patients in early stages of the disease, where small lesion in the primary tumor region are of great importance, conventional thresholding and subsequent classification reach their limitation. Interestingly, omitting small metastases for the whole-body MTV did not hamper the overall survival prediction. This might partly be explained by segmentation artifacts and partial volume effects that especially come into effect when small lesions were regarded in ^18^F-FDG PET/CT.

The study faces some limitations. It was conducted retrospectively in a single center and might therefore be affected by selection biases. Moreover, the number of included patients is relatively small, which might affect the transferability to larger patient collectives. Finally, most included cancer patients were in an advanced stage. Therefore, future study should elucidate if the whole body MTV is prognostic in earlier cancer stages as well. Given the differences in MTV_AI_ and MTV_manual_, future studies should focus on improving the segmentation of metastases with low FDG uptake.

## Conclusion

If only PERCIST measurable lesions were regarded, PARS had high accuracy in foci delineation and anatomical position determination in a cancer type it was not trained for. Likewise, PARS-derived whole-body and organ-wise MTV had good accuracy. Yet, PARS performance was much lower when dealing with all tumor foci including those manually delineated by experts. Thus, the PARS neural network seems not prone to the clever Hans effect. The automatically determined whole-body MTV is a significant prognosticator of overall survival time. The development of neural networks aiming at improved pathological FDG foci segmentation for fully automated tumor volume analysis is warranted.

## Supplementary information

ESM 1 (DOCX 12 kb)
