# Supplementary material for: Just another “Clever Hans”? Neural networks and FDG PET-CT to predict the outcome of patients with breast cancer
Source: Eur J Nucl Med Mol Imaging. 2021 Mar 5;48(10):3141–50. doi: 10.1007/s00259-021-05270-x (PMC8426242; doi:10.1007/s00259-021-05270-x)
Supplement: Supplementary file 1 — (DOCX 12 kb) [file 259_2021_5270_MOESM1_ESM.docx]

## Supplementary Table 1 – Identification and classification of suspicious FDG-avid foci by fully automated neural network when compared with the consensus readers reference standard.

|  | **Per lesion** | **Per patient** |
| --- | --- | --- |
| *Accuracy* | 69.8 (63.7 - 75.9) % | 71.5 (64.9 - 76.5) % |
| *Sensitivity* | 46.8 (37.5 - 56.0) % | 39.1 (29.8 - 49.6) % |
| *Specificity* | 97.7 (95.0 - 98.9) % | 97.6 (94.1 - 99.0) % |
| *Positive predictive value* | 96.1 (90.9 - 98.1) % | 93.8 (82.4 - 97.6) % |
| *Negative predictive value* | 60.3 (49.2 - 70.4) % | 60.8 (51.7 - 68.9) % |
| All measurable lesions were regarded for this table. | | |
